# Small Bowel Perforation due to Gossypiboma Caused Acute Abdomen

**DOI:** 10.1155/2013/219354

**Published:** 2013-10-28

**Authors:** Tahsin Colak, Tolga Olmez, Ozgur Turkmenoglu, Ahmet Dag

**Affiliations:** Department of General Surgery, Medical Faculty, Mersin University, 33079 Mersin, Turkey

## Abstract

Gossypiboma, an infrequent surgical complication, is a mass lesion due to a retained surgical sponge surrounded by foreign body reaction. In this case report, we describe gossypiboma in the abdominal cavity which was detected 14 months after the hysterectomy due to acute abdominal pain. Gossypiboma was diagnosed by computed tomography (CT). The CT findings were a rounded mass with a dense central part and an enhancing wall. In explorative laparotomy, small bowel loops were seen to be perforated due to inflammation of long standing gossypiboma. Jejunal resection with end-to-end anastomosis was performed. The patient was discharged whithout complication. This case was presented to point to retained foreign body (RFB) complications and we believed that the possibility of a retained foreign body should be considered in the differential diagnosis of who had previous surgery and complained of pain, infection, or palpable mass.

## 1. Introduction

Retained foreign body (RFB) has been reported after abdominal, thoracic, cardiovascular, orthopedic, and neurosurgical procedures [1]. RFB is a rare condition for both patient and surgeon that can lead to very serious consequences after abdominal surgery. Gossypiboma can cause serious complications such as intra-abdominal abscess in the early stages. But it might remain asymptomatic for many years. Some imaging modalities including plain radiography, ultrasonography (USG), computed tomography (CT), and magnetic resonance imaging (MRI) may help to have exact diagnosis [2]. Surgery is the recommended treatment option in these cases. Gossypiboma, in patients with a diagnosis of intra-abdominal mass and had previous history of surgery, should be considered in the differential diagnosis, even if it is a rare condition. The appropriate surgical intervention should be planned as soon as possible due to legal and medical problems. The present case had emergency hysterectomy 14 months ago and suffered from mild intermittent abdominal pain only since to present acute abdominal syndrome and was admitted to hospital. The aim of present case was to draw attention to the complication of RFB.

## 2. Case Presentation

**Figure 1:**
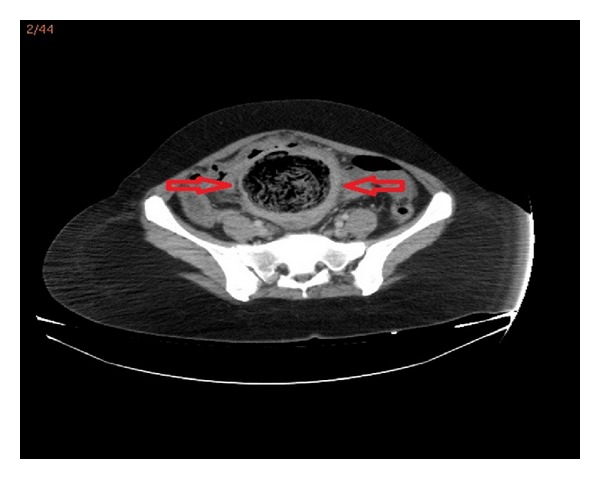
Abdominal CT scan revealed intra-abdominal mass—gossypiboma.

**Figure 2:**
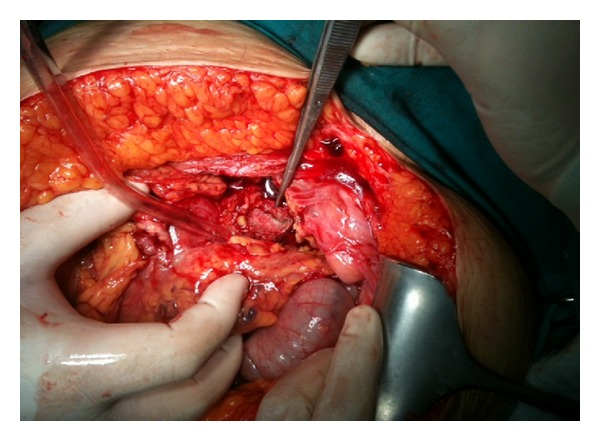
Exploratory laparotomy revealed an encapsulated sponge surrounded by omentum.

**Figure 3:**
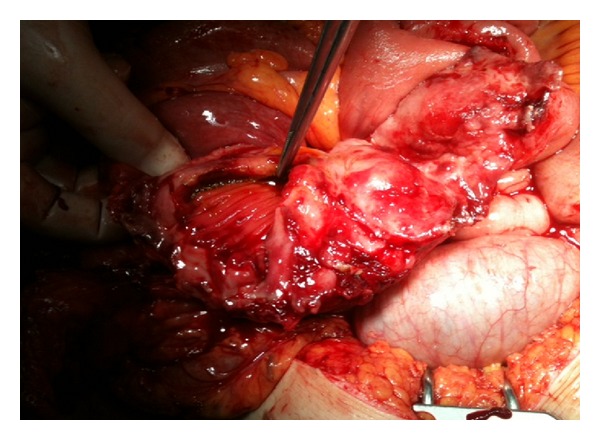
Perforation area on small bowel.

**Figure 4:**
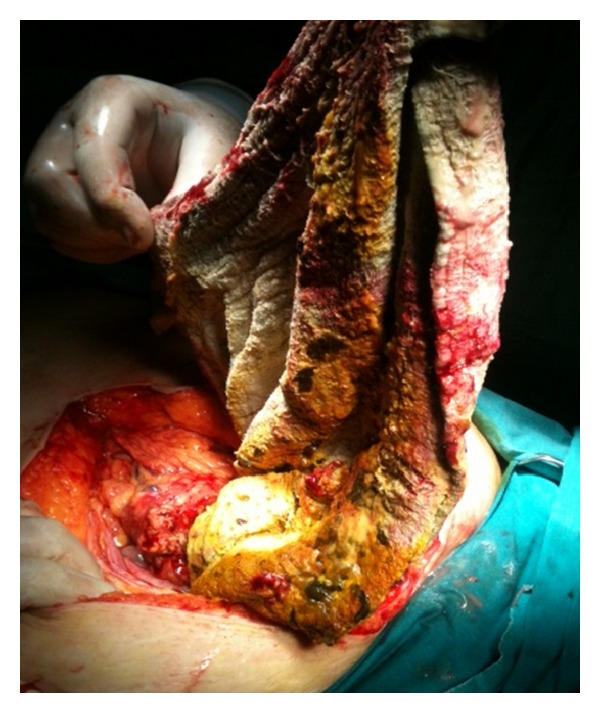
Surgical specimen (gossypiboma).

A 38-year-old woman with 27.3 BMI needed an emergency caesarean section in the fourth delivery 14 months ago in public hospital. After caesarean section, the surgeons had to perform hysterectomy due to continued bleeding. Medical record revealed no postoperative complication and the patient was discharged after one week from the hospital. In the follow up, the patient suffered sometimes from mild intermittent abdominal pain only. However, the surgeons did not perform advanced examinations and the problem was explained with postoperative adhesions. The patient was satisfied with surgeons' statements and did not continue the followup. But, after 13 months of operation, mild intermittent abdominal pain was converted to mild abdominal colic and the patient felt discomfort. Hence, last week before admittance to hospital, intermittent fever, which reached up to 39°C, and severe abdominal colic emerged. The patient was admitted to emergency department with above mentioned symptoms. Physical and laboratory examinations showed that the blood pressure was 100/80 mmHg, pulse was 110/min, body temperature was 38.5°C, WBC was 6.3 × 10^3^/*µ*L, and CRP was 241 mg/L. Abdominal USG shows a mass in pelvic area, but the source is not certain. CT demonstrated that a mass (15 × 13 cm) with a dense central part and an enhancing wall (gossypiboma) was located in the pelvic area (Figure 1). The patient underwent emergency surgery and exploratory laparotomy shows that an encapsulated abdominal compression is surrounded by omentum (Figures 2 and 3). Retained abdominal sponge caused perforation of jejunum at the 50 cm below the ligament of Treitz and was surrounded by intestinal content, which caused regional contamination (Figure 4). After removal of abdominal sponge, adhesiolysis and segmental small bowel resection with end-to-end anastomosis were performed. The abdominal cavity was irrigated with 8–10 L saline and three abdominal drains were inserted. Bowel sound was begun and serous drainage content decreased to 50 mL in postoperative day (POD) 2. The patient tolerated the solid diet at POD 3. Drains were taken at POD 5 and CRP decreased up to 15. The patient was discharged at POD 7 due to patient feeling good enough. The patient was still well in one-week and one-month followup.

## 3. Discussion

The reported incidence of retained surgical sponge is one per 1,000–15,000 abdominal operations [3]. Incidence of gossypiboma is low in developed countries due to the advanced operation room conditions and radiological techniques. A surgical sponge can be retained after any surgery but most commonly after hysterectomy, appendectomy, and cholecystectomy. Persistent wound infection, unexplained pain, and fever in the postoperative period should lead one to suspect a retained foreign body. This condition is often underestimated, because case numbers are calculated only on the basis of malpractice claims. Reason of nonreporting of occurrences is the fear of medicolegal repercussions [4]. Patients are often asymptomatic and not detected for a long time. In fact, many cases were discovered to have a retained surgical sponge more than 30 years after the initial surgery [5]. Some of patients present with abdominal mass or subacute intestinal obstruction. They may rarely result in fistula, perforation, or even extrusion per anus. In our case, the patient had also a gynecological operation 14 months ago and she had intermittent abdominal pain only. The main signs and symptoms are pain/irritation (42%), palpable mass (27%), and fever (12%) [2].

The retained surgical sponge triggers two biological responses named as aseptic fibrinous responses due to foreign body granuloma or exudative reaction leading to abscess formation [6]. The most common detection methods were computed tomography (61%), plain radiography (35%), and ultrasound (34%). So, the first diagnostic modality to rule it out should be a computed tomography scan. MRI features can be confusing because the radio-opaque marker is not magnetic or paramagnetic [7]. Intense acoustic collection in operation area or the mass can be shown by USG. If sponge contains radio-opaque marker, it can be seen in direct X-ray. The universal guideline which was stated by the American College of Surgeons in October 2005 strictly recommended that radio-opaque sponges should only be used, and accurate sponge counts should be performed before the procedure, and before and after closure of the abdomen.

Migration of retained sponge into bowel is rare but does occur when compared to abscess formation and occur as a result of inflammation of the intestinal wall that evolves to necrosis [8]. In our case, a large surgical sponge caused intestinal perforation 14 months after surgery.

Operation under emergency conditions, involvement of more than the surgical team in the operation, change in assistant staff during operation, increased BMI, volume loss, number of surgeons, and female gender are all risk factors for RFB [3]. Irrespective of the rarity of reports, operating teams should take care to count swabs used in all procedures. Surgeons should develop a habit of performing a brief but thorough routine postoperative wound and cavity exploration prior to wound closure [4]. Treatment of gossypiboma is the surgical removal usually through the previous operative site, but endoscopic or laparoscopic approaches may be attempted [9].

As a result, Gossypiboma is usually asymptomatic, has nonspecific radiological findings, and is a rare condition. These situations might delay the diagnosis. Also, a gossypiboma can cause complications such as perforation and adhesion to the adjacent structures. In order to avoid gossypiboma, the surgeons should comply with recommended statement on the prevention of retained foreign bodies after surgery. Atypical abdominal pain should be kept in mind since the gossypiboma even out of operation for a long time can be passed.
